# Numerical analysis, spectral graph theory, orthogonal polynomials and quantum algorithms

**DOI:** 10.1098/rsta.2024.0426

**Published:** 2025-10-09

**Authors:** Anastasiia Minenkova, Gamal Mograby, Hanmeng Zhan

**Affiliations:** ^1^ University of Hartford, West Hartford, CT, USA; ^2^ University of Cincinnati, Cincinnati, OH, USA; ^3^ Department of Computer Science, Worcester Polytechnic Institute, Worcester, MA, USA

**Keywords:** orthogonal polynomials, spectral graph theory, quantum computing

## Abstract

Recent progress in quantum computing shows the need to incorporate many branches of mathematics (graph theory, matrix theory, optimization, theory of orthogonal polynomials and more) into physics, computer science and chemistry. At the 2024 SIAM Quantum Intersections Convening, Bert de Jong (Lawrence Berkeley National Laboratory) gave a talk entitled ‘Quantum Science Needs Mathematicians’ (Report of the SIAM Quantum Intersections Convening. Integrating Mathematical Scientists into Quantum Research, 7–9 October 2024, Tysons, Virginia (doi:10.11337/25M1741017)), since despite the growing demand for research in these domains, the mathematical sciences community has remained largely disengaged from quantum research, with only a few isolated areas of active involvement. This issue brings together researchers from different areas of mathematics to show the relation between spectral graph theory, the theory of orthogonal polynomials and numerical analysis. This interconnectedness highlights the versatility and importance of these areas of mathematics in the context of quantum computing.

This article is part of the theme issue ‘Numerical analysis, spectral graph theory, orthogonal polynomials and quantum algorithms’.

## 1. Orthogonal polynomials, perfect state transfer and other quantum mechanics problems

For one-dimensional chains, the Hamiltonian is given by a Jacobi matrix, and the mathematical aspect of the problem of perfect quantum state transfer is that there is a symmetry ‘the perfect transfer’ condition on the eigenvalues of the matrix in question. Since a Jacobi matrix defines a sequence of orthogonal polynomials, this condition allows us to get a characterization of Jacobi matrices realizing the perfect state transfer [1].

This insight has spurred further research into the relationship between orthogonal polynomials and quantum state transfer. For more information, see [2,3].

The mathematical structure of quantum mechanics consists of Hilbert space over the complex field. Significant research has been dedicated to extending quantum mechanics beyond the complex field to address various emerging challenges. Birkhoff and von Neumann introduced the field of quaternions to represent the pure states of a quantum system on any associative division algebra. The study of orthogonal polynomials over bicomplex numbers has emerged as a viable alternative [4].

## 2. Spectral graph theory and orthogonal polynomials

There are deep connections between graph theory and orthogonal polynomials. For example, computing the characteristic polynomials of the adjacency matrices of certain graphs generates orthogonal polynomial sequences such as Hermite polynomials, Chebyshev polynomials and others [5].

Applications of quantum computing enriched the research of both areas of mathematics. The state-transfer related problems that we mentioned before could be extended to the inverse problems for Laplacian or adjacency matrices of graphs. Hence, one could translate the polynomial language to matrices and then to graphs associated with a quantum system. The connection becomes more evident through the prism of the quantum walks. Some quantum walks can be modelled using weighted graphs, where each vertex represents a qubit, and each weighted edge represents the coupling strength between two qubits. The work that is done in this direction involves graph theory, orthogonal polynomials, quantum probability, matrix analysis, numerical analysis, scattering theory and many more areas of mathematics and physics. For more information, see [6].

## 3. Numerical analysis of quantum algorithms

The physics of quantum computers brings the need to change the classical algorithms and to find new approaches. Shor’s and Grover’s algorithms are the most famous examples of quantum algorithms outperforming their classical counterparts. The study of these algorithms involves working with graph-related matrices like Laplacians and the study of their spectral data, where the properties of orthogonal polynomials come in handy. For more information, see [7].

It is important to note that our previous discussion can be viewed through the lens of structured matrices or Hilbert spaces involved in quantum computing. From Jacobi matrices to Hermitian and unitary matrices (under a suitable Hilbert space), and concluding with positive matrices, each part leverages the properties of these matrices to achieve their respective results.

## 4. The content of this issue

Our theme issue collects 10 contributions, including two review papers and eight original research papers. It integrates all three branches of mathematics, we discussed and establishes their connections to quantum computing.

We start with papers dedicated to orthogonal polynomials. The first contribution is the review paper by R. Bailey, which explores applications of orthogonal polynomials in quantum information processing, focusing on Jacobi operators for detecting perfect state transfer and drawing analogies to classical birth–death processes via Karlin and McGregor’s results. It also introduces extensions to quantum walks with more than nearest-neighbour interactions using exceptional orthogonal polynomials, while offering a concise, self-contained introduction and providing some open questions.

P.-A. Bernard & L. Vinet discuss symmetric quantum n-qubit states, such as Dicke states, which play a key role in quantum information, prompting interest in efficient methods for their preparation and manipulation. This work presents two simple transformation protocols, characterizes their algebraic structure using the Weyl algebra W(2) and su(2), identifies their fixed points and explores connections to the binary Hamming scheme, Hadamard transform and Krawtchouk polynomials.

D. Alpay *et al.* extend complex C-R calculus and Hermite polynomials to the bicomplex setting, comparing the resulting function theory with the classical case. This work introduces two types of bicomplex Hermite polynomials, explores their properties and examines the action of three bicomplex Landau operators on them.

Motivated by the product formula of Chebyshev polynomials of the second kind, W. Mlotkowski and N. Obata introduce partial Chebyshev polynomials and derive their properties and new factorizations.

The next block of contributions focuses on the relationship between graph theory and quantum computing. Pretty good state transfer between the antipodal vertices of the hypercube $Q_{d}$ was known for prime d for the arc-reversal walks with Grover coins. H. Zhan generalizes this by designing real, weighted Grover coins for all d, which require modifying the weight on one arc per vertex.

Chiral quantum walks offer a promising way to bypass no-go theorems in quantum transport but remain unexplored. A. Acuaviva *et al.* prove that universal monogamy violations require more than imaginary couplings, leading to new constructions on sparse graphs and the first one-way perfect transfer using transcendental couplings.

B. Maloy *et al.* analyse Grover’s quantum walk on segmented complete graphs, which blend symmetry with rich spectral features. This extends classical results and offers new ways to improve quantum search on complex networks. This work is a good transition from graph theory to the numerical analysis part.

The next contribution is by V. Borovyk & B. Vellabi. Distinguishing quantum states and channels is key in quantum computing and information theory. This review paper compares various metric-based approaches, highlighting their goals, properties and trade-offs.

R. Devendra *et al.* study quantum Gaussian channels which are key to continuous-variable quantum communication. This work unifies the definitions and explores their physical realization via linear optics. The authors introduce new characterizations, answer open questions and clarify common misconceptions in the literature.

Finally, S. De *et al.* consider variational quantum and neural quantum states algorithms for the linear complementarity problem. Variational quantum algorithms aim to harness quantum advantages on today’s noisy hardware, but their real-world effect is still uncertain. The authors apply both quantum and quantum-inspired solvers to simulate rigid-body collisions, showing they can rival classical methods in physical modelling.

The editors of this thematic issue extend their heartfelt thanks to all the authors for their valuable contributions. They also express sincere appreciation to the entire editorial team of *Philosophical Transactions A* for their indispensable support in bringing this thematic issue to fruition.

## Data Availability

This article has no additional data.
